# A Cost-Effective Method for Automatically Measuring Mechanical Parts Using Monocular Machine Vision

**DOI:** 10.3390/s23135994

**Published:** 2023-06-28

**Authors:** Vinicius V. E. Nogueira, Luiz F. Barca, Tales C. Pimenta

**Affiliations:** 1 Federal Institute of Paraná, Campo Largo 83607-140, Brazil; 2 Institute of Systems Engineering and Information Technology, Federal University of Itajubá, Itajubá 37500-903, Brazil; tales@unifei.edu.br; 3 Institute of Mechanical Engineering, Federal University of Itajubá, Itajubá 37500-903, Brazil; barca@unifei.edu.br

**Keywords:** computer vision, close-range terrestrial photogrammetry, monocular vision, planar measurement

## Abstract

Automatic measurements via image processing can accelerate measurements and provide comprehensive evaluations of mechanical parts. This paper presents a comprehensive approach to automating evaluations of planar dimensions in mechanical parts, providing significant advancements in terms of cost-effectiveness, accuracy, and repeatability. The methodology employed in this study utilizes a configuration comprising commonly available products in the industrial computer vision market, therefore enabling precise determinations of external contour specifications for mechanical components. Furthermore, it presents a functional prototype for making planar measurements by incorporating an improved subpixel edge-detection method, thus ensuring precise image-based measurements. The article highlights key concepts, describes the measurement procedures, and provides comparisons and traceability tests as a proof of concept for the system. The results show that this vision system did achieve suitable precision, with a mean error of 0.008 mm and a standard deviation of 0.0063 mm, when measuring gauge blocks of varying lengths at different heights. Moreover, when evaluating a circular sample, the system resulted in a maximum deviation of 0.013 mm, compared to an alternative calibrated measurement machine. In conclusion, the prototype validates the methods for planar dimension evaluations, highlighting the potential for enhancing manual measurements, while also maintaining accessibility. The presented system expands the possibilities of machine vision in manufacturing, especially in cases where the cost or agility of current systems is limited.

## 1. Introduction

Mechanical parts manufacturers have increasingly stringent requirements for tolerances in their specifications. Meeting these demands means more extensive testing of parts to optimize machine setup parameters, and to ensure quality control [1]. Meticulous manual measurement techniques demand the operator’s attention and precision. Furthermore, the samples must be randomized. This together results in a time-consuming process [2].

The application of image processing for automatically making measurements can enhance the efficiency of this process by allowing more parts to be measured within the same time frame, while also mitigating measurement discrepancies that can result from human error [3]. One approach for automating measurement processes involves integrating computer vision and photogrammetry techniques.

Vision plays a crucial role in human perception, allowing individuals to interpret and comprehend various aspects of the surrounding world [4]. Computer vision is a field that seeks to replicate this ability in machines to enhance their capabilities. Computerized analysis facilitates a faster and more precise extraction of information through image examination [5]. Consequently, the use of related methodologies ensures repeatability and reliability in tasks demanding high precision.

Among the distinctive features of this field, the absence of physical contact between the equipment and the analyzed sample should be highlighted. Moreover, many different dimensions of the analyzed object can be captured within a single image, even for irregularly shaped objects [6].

Photogrammetry involves deriving three-dimensional geometry from photographic images [7,8]. Photogrammetry has found successful applications in various domains, e.g., for unmanned aerial vehicles and satellite mapping [9], bridge monitoring [7], three-dimensional object reconstruction [10], 3D scene mapping [11], and precise object measurements [3,12,13,14,15,16,17]. This field of study has versatile applications in many industries including geology, architecture, topography, automotive engineering, and aerospace [8].

In recent years, the field of photogrammetry has undergone significant advancements. These advancements can be attributed to the progress of digital image capture technologies, which have reduced costs and increased equipment capabilities. Furthermore, developments in related areas, e.g., improved machine and software processing power, have also contributed to progress in the photogrammetry field. Consequently, previous limitations associated with this technique have since been overcome, generating increased interest in this field, given its agility, versatility, and appropriate level of precision [9,18].

Multiple methods exist for extracting measurements of an element from images, ranging from techniques based solely on monocular vision to those employing the triangulation of multiple elements, e.g., Stereoscopic Vision [11] and Pattern Projection [10].

Techniques that solely rely on monocular geometry necessitate a controlled environment and additional reference information, making them more application-specific [19,20,21,22]. Nevertheless, monocular techniques offer a cost-effective solution in that they require fewer devices, and are still quite accurate [12].

Previous studies have addressed object dimension measurement using Monocular Machine Vision, with various applications such as measuring critical dimensions in LCDs [14], evaluating the edges of heat-emitting metals [15], measuring gears [13], measuring elements on the contours of ring-shaped objects [3], and measuring shafts [12,16,17].

The success of measurements through images hinges upon the precise localization and mapping of points of interest from the image to the real world. However, the accuracy with which these points of interest can be located is influenced by the specific characteristics of the image acquisition equipment, e.g., the resolution, noise levels, and image artifacts. To overcome these challenges, this study employs subpixel edge-detection techniques and noise reduction methods on images obtained from a monochromatic camera. Additionally, mapping between image coordinates and the real world was achieved via planar checkerboard calibration patterns, which established the measurement plane and captured characteristics based on the Projective Camera model.

This significant contribution of this paper is that it introduces an alternative method for subpixel edge detection. The technique presented herein improves point localization via regression and precise angle estimations. This approach uses a modified one-dimensional edge model for the regression in the discretized edge direction. The result is then adjusted to the normal direction, and projected into 3D space, enabling a second regression based on neighboring points. Accurate estimations of the normal direction for the analyzed edge pixel are pivotal in this process. To address this, this article further proposes employing a Kroon filter [23] in conjunction with a zero-phase filter. Lastly, this paper outlines the workflow for the applied subpixel localization method, offering a comprehensive overview of the employed techniques along with a detailed procedure outline.

This article also highlights its noteworthy contributions in the form of a functional prototype. The description of the device developed herein includes details regarding the component selection, along with the adopted strategy and operational conditions. Regarding the prototype’s operation, this article discusses implementing computer vision and photogrammetry techniques, the problem formulation, the limitations of the monocular measurement technique with regular lenses, and provides an operational flowchart.

A series of tests were conducted in a Metrology Laboratory to assess the performance of the device. First, a traceability test was performed, by measuring gauge blocks. Additionally, a comparative test was carried out against a Coordinate Measurement Machine, using a cylindrical sample as the object of evaluation. The results from the traceability tests revealed a mean error of 0.008 and a standard deviation of 0.0063. These values were subsequently compared with existing research in the field, validating the effectiveness and accuracy of the device developed here.

The primary objective of this device is to streamline and standardize the measurement process. It employs a monocular vision technique with readily available components, such as the IMX178, an industrial entry-level sensor, regular low-distortion 16 mm lenses, and diffuse backlighting. The devices chosen enhance cost-effectiveness and accessibility for procurement and replacement. The proposed solution aims to enhance the feasibility of measuring the outer contour of mechanical parts under given conditions, without requiring telecentric lenses or additional equipment for conducting the photogrammetric techniques.

## 2. Materials and Methods

### 2.1. Monocular Geometry

The camera model establishes the correspondence between a point in space and its representation in an image. This relationship relies on knowledge of the capture set parameters and the pose of the measurement plane with respect to the camera. When a camera’s characteristics are determined, it is referred to as a calibrated camera. During the image formation process, or image projection, the camera matrix incorporates the perspective phenomenon and accounts for misalignment between axes in homogeneous coordinates, based on the pinhole model as defined in Equation (1) [24].

(1) $$\begin{array}{c} \left[\begin{array}{c} x_{h}\\ y_{h}\\ z_{h} \end{array}\right]=\left[\begin{array}{ccc} c_{x} & 0 & p_{x}\\ 0 & c_{y} & p_{y}\\ 0 & 0 & 1 \end{array}\right]\left[\begin{array}{ccc} \begin{array}{ccc} R_{11} & R_{12} & R_{13}\\ R_{21} & R_{22} & R_{23}\\ R_{31} & R_{32} & R_{33} \end{array} & | & \begin{array}{c} t_{1}\\ t_{2}\\ t_{3} \end{array} \end{array}\right]\left[\begin{array}{c} X\\ Y\\ Z\\ 1 \end{array}\right] \end{array}$$

$$x^{h}=K\;\left[R|t\right]\;X^{h}$$

In this Projective Camera model, the K camera matrix performs the perspective transformation. The camera constant c reproduces the focal length on each axis and pixel size, while the principal point p indicates the location where the optical axis intersects the image.

The adequacy of the difference between the world and camera axes is assessed by a rigid body transformation, where *R* is the rotation matrix and *t* is the translation matrix. In this transformation, the origin of the camera axes *C* is implicitly expressed in the translation *t*, through the relation t=−RC [25]. Libraries such as OpenCV widely adopt this implicit representation model to express image projection models.

The inverse projection, which maps image coordinates to real-world coordinates, can be derived by inverting the image projection matrices [26]. However, the reverse process does not yield a single solution, but rather a set of solutions. The spatial location of a point can be determined relative to the object’s depth with respect to the camera. This ambiguity is represented by a scalar λ in the input vector, present in the homogeneous coordinate model. The set of solutions is described by a straight line intersecting the camera origin point and the corresponding point in the image. Consequently, through manipulations and mathematical properties, the inverse projection process can be described in Equation (2):

(2) $$x^{h}=\lambda x^{h}\;X_{w}^{h}=R^{T}\;K^{-1}\;\lambda x^{h}-R^{T}t$$

The use of a lens in an image capture system significantly reduces the exposure time by improving the capture of light rays. However, the lens introduces distortions to the image, shifting information from one point to another, even when high-quality lenses are employed. These distortions depend on the physical characteristics of the lens assembly. Once this phenomenon is corrected in the image, as the pinhole model accurately represents image formation [25].

The lens distortion model, as depicted in Equation (3), comprises two main types of distortions: radial distortion and tangential distortion. Both types of aberrations are minimal at the principal point of the image, and increase as one moves away from this point, reaching a maximum at the image edges [12].

(3) $$\begin{array}{cc} D_{x}\left(x_{i},y_{i}\right) & \approx x_{i}\left(1+k_{1}r^{2}+k_{2}r^{4}\right)+\left[2p_{1}x_{i}y_{i}+p_{2}\left(r^{2}+2x_{i}^{2}\right)\right]\\ D_{y}\left(x_{i},y_{i}\right) & \approx y_{i}\left(1+k_{1}r^{2}+k_{2}r^{4}\right)+\left[2p_{2}x_{i}y_{i}+p_{1}\left(r^{2}+2y_{i}^{2}\right)\right] \end{array}$$

In this model, positions $x_{i}$ and $y_{i}$ represent the coordinates of a point in the undistorted image, which are shifted, due to distortion, to positions $D_{x}$ and $D_{y}$. The variable r corresponds to the distance from the analyzed point to the main point. Parameters $k_{1}$ and $k_{2}$ are estimated parameters for radial distortion, while $p_{1}$ and $p_{2}$ refer to tangential distortion.

Therefore, the calibration process is crucial for precise measurements and involves computing the values of the *K*, *R*, and *t*, in addition to the lens distortion parameters. The matrix parameters *K* and the distortion parameters are intrinsic to the capture set characteristics (camera and lens). On the other hand, the extrinsic parameters refer to the *R* matrix and the *t* vector, which depend on the arrangement of the capture set relative to the reference plane.

**Figure 1:**
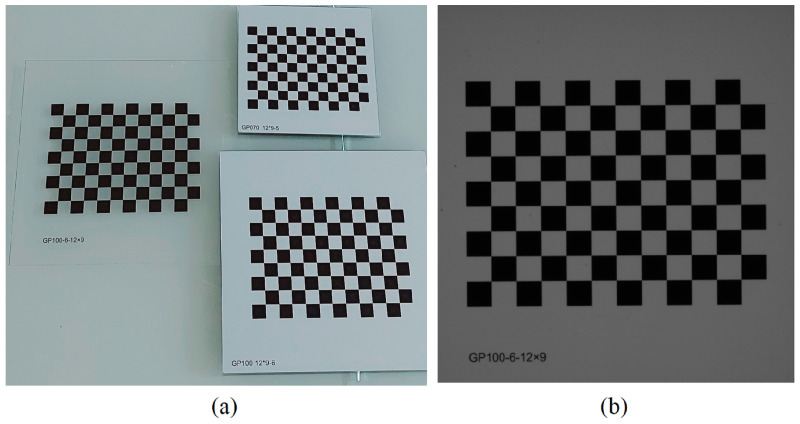
Calibration patterns used for estimating intrinsic and extrinsic parameters (**a**) Checkerboard calibration patterns with a tolerance of 0.01 mm (**b**) Image of the translucent pattern placed on the measuring area captured by the camera in the working position to calibrate the extrinsic parameters and establish the base plane.

Several techniques exist for calibrating the intrinsic parameters of cameras, primarily based on a procedure defined by [27]. In this procedure, intrinsic calibration is performed using a planar calibration pattern, as shown in Figure 1a. The algorithm, implemented using the OpenCV library, computes the intrinsic parameters based on a set of images of the calibration pattern for different poses.

During the calibration of extrinsic parameters, rotation and translation of the base plane with respect to the camera are estimated using a checkerboard calibration pattern and the previously estimated intrinsic parameters. The pattern is positioned over the measurement area (Figure 1b) and mapped to the 3D world using reference points extracted from the image. For this purpose, the Perspective-n-Point Camera Pose Determination (PnP) algorithm, included in the OpenCV library, is employed. This algorithm effectively calculates the extrinsic parameters (*R* and *t*) based on the mapped points [26], compensating for tilt and distance. Following this procedure, the surface of the calibration pattern serves as the reference for the base plane, and is excluded from the prototype during operation. In Section 2.4 we discuss the procedure for determining the 3D projection given the intrinsic, and extrinsic parameters, and measuring height relative to the calibrated plane.

### 2.2. Kroon Filter

Kroon [23] proposed a kernel for performing edge filtering, specifically designed to address deviations in sampled angles observed in conventional filters such as Sobel and Scarr. These deviations become more pronounced as the direction of the analyzed edge moves away from the sampled directions. The configuration presented in Equation (4) represents a numerical optimization-based arrangement that minimizes the absolute angle error.

(4) $$\begin{array}{c} H_{x}=\left[\begin{array}{ccccc} 0.0007 & 0.0052 & 0.037 & 0.0052 & 0.0007\\ 0.0037 & 0.1187 & 0.2589 & 0.1187 & 0.0037\\ 0 & 0 & 0 & 0 & 0\\ -0.0037 & -0.1187 & -0.2589 & -0.1187 & -0.0037\\ -0.0007 & -0.0052 & -0.037 & -0.0052 & -0.0007 \end{array}\right] \end{array}$$

**Figure 2:**
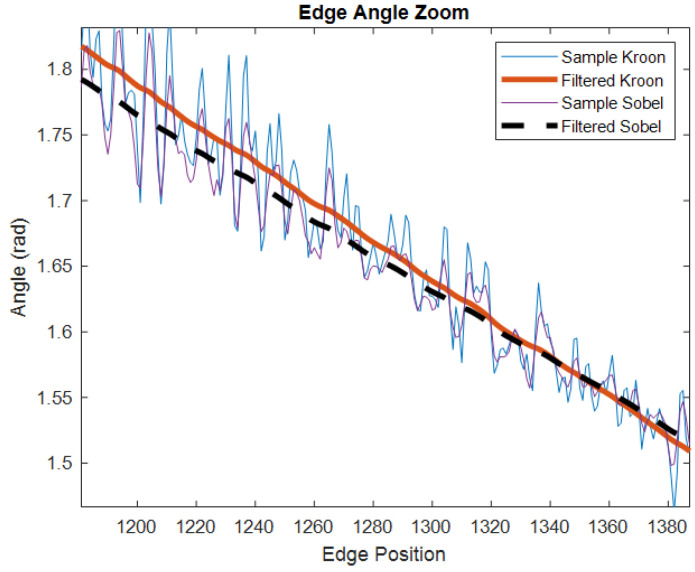
A comparison between the Sobel and Kroon filters for estimating edge orientation on a section of a circular sample.

The kernel was used in this study to assist in extracting points of interest and accurately locating them within the pixel. To further enhance accuracy, a zero-phase lowpass filter was applied to the contour to reduce sampling noise along the edges, as illustrated in Figure 2.

### 2.3. Subpixel Edge Detection

Subpixel edge-detection techniques allow for the evaluation of edge positions within pixel regions with high precision. The technique involves extrapolating the edge profile using mathematical models, therefore determining the edge positions based on model-sampled pixel values.

Most methods can be categorized into three groups: moment-based, interpolation-based, and regression-based. Moment-based models are effective under low noise levels; however, they are more susceptible to noise due to the use of numerical differentiation [12]. Interpolation-based methods are less affected by noise compared to moment models, but they yield less accurate results. Regression-based methods, also known as reconstructive methods, utilize optimization algorithms to calculate optimal model parameters through error minimization. This approach attenuates the influence of noise. However, regression-based methods require more computational resources compared to the other two methods [28].

The edge model used in this study considers the blurring effect on edges caused by optical limitations, image formation processes, or filtering. In this model, the edge exhibits a sigmoidal shape. Various models, such as the hyperbolic tangent function [29], arc tangent [12], a Gaussian integral function [13,28], or logistic function [14] can be used to describe the sigmoidal form of the edge. Similarly, the authors in [15] perform regression on the first derivative of the edge, modeling it using the Gaussian function. However, this technique has the drawback of amplifying the influence of noise due to the use of the first derivative.

Since images are two-dimensional, one-dimensional models only consider samples oriented in the discretized direction perpendicular to the edge, since this direction exhibits the most significant gradient variation. Yu [28], proposes a two-dimensional estimator based on the edge shape, while [12] performs one-dimensional regression and subsequently applies a correction based on the estimated angle. The authors in [15,30] perform regression at multiple points along the edge, using the points found in a subsequent regression process with a model based on the edge’s shape.

By contrast, this paper employs a modified logistic function as a one-dimensional model for the edge, as represented in Equation (5).

(5) $$T\left(x\right)=\frac{p_{2}-p_{1}}{1+10(p_{3}-x)p_{4}}+p_{1}$$

**Figure 3:**
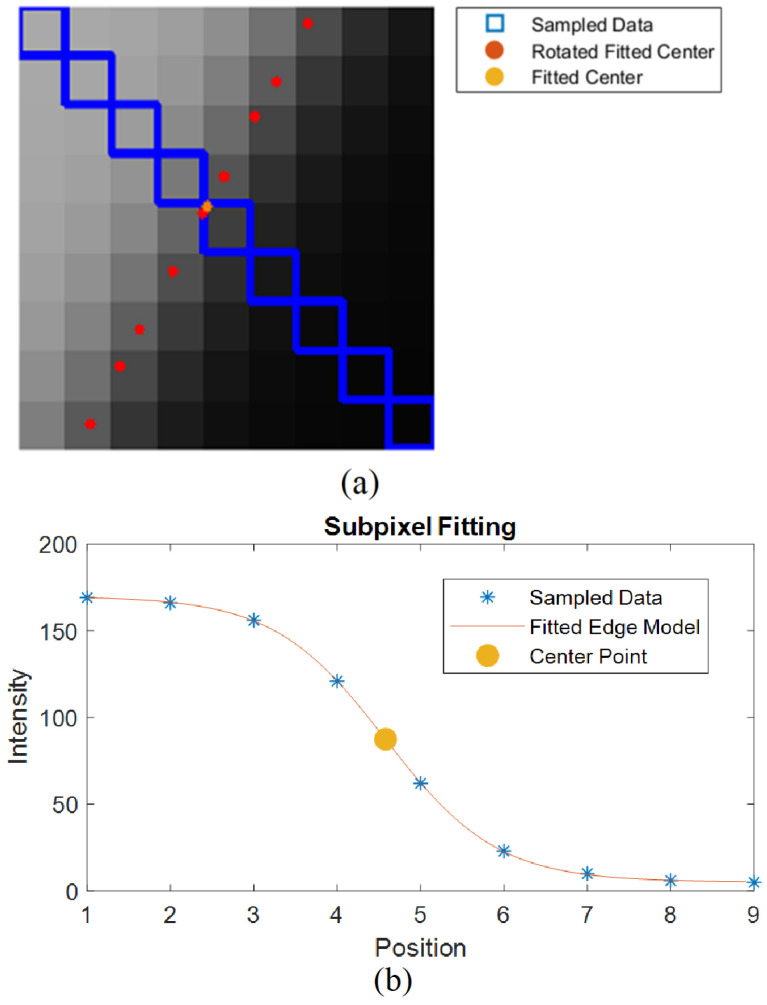
Subpixel Edge Detection applied to an edge with a normal orientation at −29.88°, discretized at 135° direction. The fitting method resulted in, $p_{1}=169.76$, $p_{2}=5.21$, $p_{3}=4.58$ and $p_{4}=0.65$. The fitted position is further rotated in the normal direction. (**a**) Representation of the subpixel estimation process on section of the contour (**b**) Fitted Edge Curve in Sampled Data.

In this sigmoidal function, most of the parameters directly refer to the curve characteristics. Variable $p_{1}$ determines the asymptote that limits the initial point value, $p_{2}$ determines the asymptote that limits the final point value, $p_{3}$ determines the central position of the curve, and $p_{4}$ determines the slope of the curve, which is $p_{4}>0$. Thus, the edge position can be directly determined from the value of the $p_{3}$ variable, by applying a nonlinear least squares regression on the sampled points, as shown in Figure 3.

As proposed by [12], after regression in the discretized direction of the maximum gradient, the position is then rotated from the discretized orientation to the direction of the edge’s normal line, as shown in Figure 3a. Therefore, reliable angle estimation is crucial for accurate edge projections, and refined angles are thus used. In [12], the angle is enhanced by regressing neighboring pixels and extracting the normal direction. In contrast, this study proposes using filtered angles obtained through the Kroon filter and zero-phase filter. This proposed enhancement mitigates errors arising from perspective-induced shape distortion and discrepancies between the regressed shape and the actual contour segment shape.

Similar to [15,30], the proposed system performs additional regression between neighboring edges using appropriate functions. However, instead of directly performing regression on the image, the points are first projected back into the world. This approach prevents potential distortions caused by lenses or perspectives from affecting the shape between neighbors. Similarly, the preprocessing operation of the entire image to correct estimated lens distortions is limited to correcting the points of interest during the inverse projection.

The Levenberg-Marquart method was applied in this project to estimate the parameters of the edge equation by fitting the curve to the sampled values. As depicted in Figure 3a, the sampled values are the pixel values in the direction normal to the edge, with the edge pixel located at the center. Since nonlinear regression is susceptible to local minima, the initial estimation parameters were strategically defined as follows: $p_{1}$, represents the value of the first sampled point, $p_{2}$ corresponds to the value of the last sampled point, $p_{3}$ denotes the integer position of the central element in the window, and $p_{4}$ is assigned a value of 0.5. These initial parameters facilitate effective convergence toward the solution.

### 2.4. Problem Formulation

The photogrammetry technique employed in this study was monocular geometry. As discussed earlier, this approach offers cost-effective advantages compared to other techniques in the field, but it relies on specific problem assumptions. Hence, certain premises must be established to ensure the convergence of the problem towards a solution.

In the monocular geometry strategy presented here, all measurements were referenced to the calibration pattern’s surface on the reference plane.

**Figure 4:**
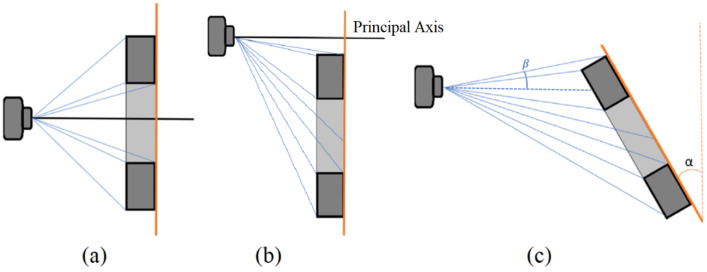
System Assumptions. Capture scheme with an optical center (**a**) inside the part, (**b**) outside the part, and (**c**) with an excessive inclination of the part. Arrangements (**b**,**c**) should be avoided.

The proposal is based on the premise that measurements are performed on the external contour of the object, and these measurements are planar, meaning that all measured points are at a known height Δz. With these assumptions, precautions can be established to ensure proper measurements, as illustrated in Figure 4.

As shown in Figure 4, if the sides of the object are visible in the image, the measurement can be performed at a different height than the intended one. However, two precautions should be taken to prevent this: first, the main point of the image should be located between the outer edges (Figure 4b), and the inclination of the object’s plane must not exceed the inclination of the ray reaching the point of interest (Figure 4c). To address this problem, the edge of the object should be moved away from the main point, and reasonable skews, which are found during the intrinsic and extrinsic calibration, should be employed. Considering that the monocular setup cannot distinguish the depth of the sampled point, these considerations are crucial.

The inverse projection equation can be used to determine the spatial location of a point based on the thickness (as height) Δz of the object and the thickness Δh of the planar calibration pattern, which serves as a reference.

The method involves first determining the scaling factor value and subsequently calculating the coordinates in the world $X_{w}^{h}={\left[X\;Y\;Z\right]}^{T}$.

According to Equation (2), where $x^{h}={\left[x_{i}\;y_{i}\;1\right]}^{T}$ represents the coordinates of the point in the image after lens distortion correction, the components $R^{T}K^{-1}x$ and $R^{T}t$ are vectors (3 × 1) and their formats can be represented with Equation (6):

(6) $$\left[\begin{array}{c} a_{1}\\ a_{2}\\ a_{3} \end{array}\right]=R^{T}K^{-1}x\;\left[\begin{array}{c} b_{1}\\ b_{2}\\ b_{3} \end{array}\right]=R^{T}t$$

Thus, Equation (2) can be rewritten as Equation (7).

(7) $$\left[\begin{array}{c} X\\ Y\\ Z \end{array}\right]=\left[\begin{array}{c} a_{1}\lambda -b_{1}\\ a_{2}\lambda -b_{2}\\ a_{3}\lambda -b_{3} \end{array}\right]$$

Employing Equation (8), the value of the scalar λ can be determined based on prior knowledge of *Z*, where *Z* corresponds to the height of the measured point relative to the reference plane, defined as Z=−(Δz−Δh).

(8) $$\lambda =\frac{b_{3}+Z}{a_{3}}$$

Finally, the three-dimensional coordinates in space can be computed using Equation (7). The accuracy of the obtained coordinates depends on the precision of the camera calibration, the point’s location in the image, and various factors e.g., camera characteristics, lens quality, lighting conditions, calibration patterns, and the computer vision algorithm used to locate points in the image.

**Figure 5:**
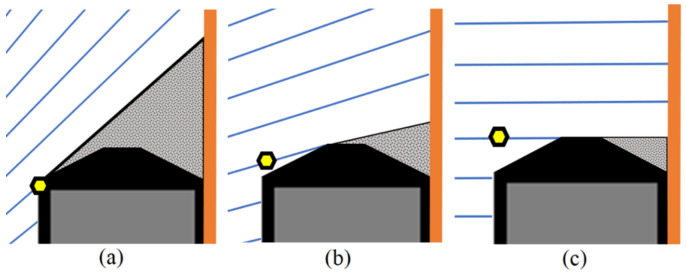
Edge profile occlusion related to focal length. Analysis of the sampled piece using regular lenses of (**a**) low focal length, (**b**) higher focal length, and (**c**) telecentric lenses.

The approach employed assumes that the sides of the object, at a height different from the one stipulated, are not visible in the image. Therefore, imperfections in the object’s finish, conicity, or chamfers can distort its contour, and lead to deviations in the estimations, as shown in Figure 5.

Figure 5a shows the expected occluded region, where the side is not visible in the image. The size of this occluded region depends on the inclination of the captured light rays, which is associated with the focal length and position in the image. Thus, the more parallel the rays, the better the observation and measurement accuracy of the edge aspects.

It is important to note that even when a feature is visible to the camera, the position of the edge may deviate from its true location if there is a difference between the assumed Δz and the actual measurement depth, as shown in Figure 5b. The more parallel the captured rays, the less this divergence will affect the measurement accuracy, as shown in Figure 5c.

Increasing the focal length can reduce the deviation by producing less inclined rays. However, this also results in longer working distances for the same field of view and stricter alignment requirements between the measurement plane and the camera (Figure 4c). Therefore, selecting the appropriate focal length is crucial for ensuring accurate measurements.

Alternatively, context-specific corrections can be applied to enhance measurement accuracy when dealing with parts that have similar characteristics. Ultimately, careful consideration of the trade-offs and limitations associated with regular lenses is necessary when considering their use in measurement applications.

### 2.5. Setup

**Figure 6:**
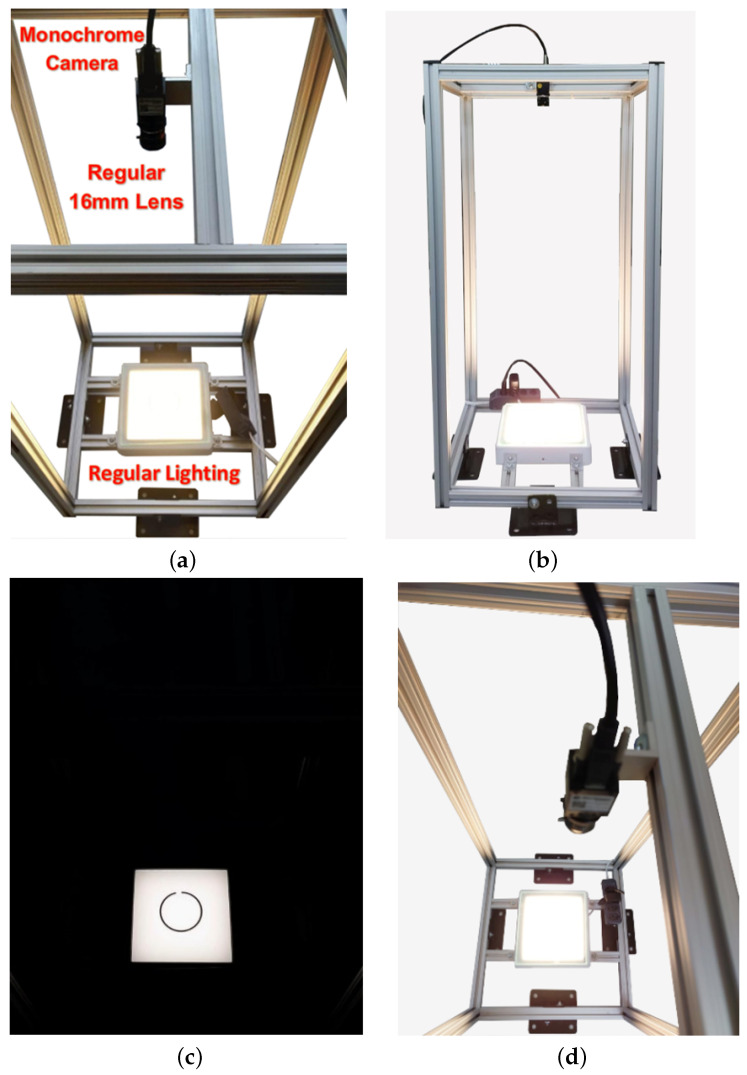
Physical configuration of the prototype. (**a**) Overview, (**b**) top view, (**c**) sample-focused view and (**d**) top view.

The employed configuration, as shown in Figure 6, employs an aluminum profile structure with the lighting positioned at the bottom and the camera positioned 0.8 m above it. The presented solution uses more affordable equipment that is readily available on the market.

Instead of using telecentric lenses, the prototype employs 16 mm fixed focal length lenses. Telecentric lenses could have been used to capture parallel light rays and simplify the measurement problem by eliminating perspective. However, telecentric lenses are more expensive given their complex construction, larger size, and stricter specifications. Additionally, in static environments, these lenses demand that the lens area be equal to or larger than the object’s dimensions, which increases costs based on the object’s size. Therefore, ordinary lenses, such as fixed-length and varifocal lenses (zoom lenses) are suitable for projects that do not require telecentric lenses, given the lower cost, ready availability from multiple suppliers, and general market availability.

A backlighting lighting scheme was employed, where the sample is positioned between the light source and the camera. This setup operates on the principle that an opaque object blocks the direct passage of light, resulting in high contrast between the background and the measured object. It also offers greater protection from ambient light given the predominant luminous flow from the light source. Additionally, it reduces exposure time and prevents glare, which is common to mechanical parts with metallic surfaces.

When selecting the image sensor, the size of individual pixels plays a significant role in capturing light. Larger pixel dimensions result in improved light-gathering capabilities, leading to better image quality [31]. Thus, larger pixels require less amplification to produce optimal image values, therefore maintaining a high signal-to-noise ratio. To ensure minimal noise and high accuracy in detection outcomes, it is crucial to apply minimal amplification, since increasing gain adversely affects the signal-to-noise ratio.

In general, sensors with larger pixel sizes, even with identical resolutions, often come at a higher cost due to their increased physical dimensions. The IMX 178 sensor (Sony, Tokyo, Japan) integrated into the MV-SUA630M camera (Medvision, Tampa, FL, USA) is an entry-level sensor with 2.4 × 2.4 μm pixel size. It embodies certain characteristics that facilitate light capture. The sensor’s back-illuminated structure exposes the photosensitive element directly to incident light, optimizing photon capture and dynamic range within CMOS sensors.

Another advantage of this sensor is its monochromatic pixels, which offer advantages such as enhanced light capture and can avoid image artifacts. Unlike color sensors, the monochromatic variant receives a higher inflow of light given the absence of color filters. Additionally, grayscale pixels extract information directly from their corresponding regions, whereas color pixels possess only a single-color filter dictated by patterns such as the Bayer pattern. Consequently, color sensors require interpolation of nearby pixels through Demosaicing techniques to infer the remaining colors [32]. These techniques can introduce artifacts into the image. Thus, in applications where color is not needed, monochromatic cameras are advantageous.

Additionally, this study used a variety of strategies aimed at reducing image noise. The backlighting setup employed emitted a significant luminous flow from the light source, directly illuminating the sensor. In addition to augmenting the contrast, this type of illumination allows the sensor to function with minimum amplification. Furthermore, the static nature of the suggested measuring equipment allows for image stacking, an effective technique for reducing noise. Consequently, multiple images are averaged to yield a noise-reduced image. In addition, a Gaussian filter was deployed to counteract image noise.

Regarding sensor resolution, it is generally accepted that increased resolution equates to enhanced detail. To overcome the inherent restrictions associated with the sensor’s resolution, subpixel-level localization was employed. This process involves model fitting along the edge direction and adjacent pixels. The result is that edge location is not limited by the sensor’s resolution, but is further extended to precisely detect edges within the individual pixel regions.

Thus, the current study seeks to fully exploit the potential of the sensor and guarantee satisfactory system performance.

### 2.6. Measurement Procedures

The software component of the equipment was developed using the C++ language, supported by the OpenCV library for computer vision and the graphical interface facilitated by Qt. The implemented algorithm is designed to accurately extract points of interest from the image, execute conversions to real-world representations, and ultimately perform measurements in physical dimensions. This process starts with the application of computer vision techniques, progresses through the refinement of estimates via nonlinear models, and culminates in the use of photogrammetry to map image points to real-world coordinates.

**Figure 7:**
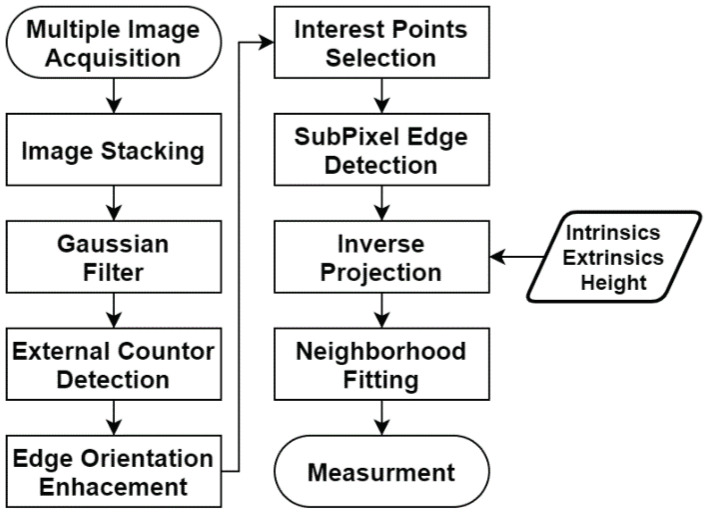
Measurement process diagram.

Figure 7 shows the measurement algorithm. It operates on the assumption that the camera is correctly adjusted and that both the intrinsic and extrinsic parameters have been previously calibrated.

1. **Image Capture:** Acquire multiple images.
2. **Image Stacking:** Merge several static images to create a single image with reduced noise. The alignment of images is unnecessary given the absence of movement between them, which simplifies the process. The average of the images is computed to produce the stacked result.
3. **Gaussian Filter:** Apply a Gaussian filter to decrease noise and facilitate edge detection.
4. **External Contour Detection:** Implement pixel-level edge detection in the image to filter the information using techniques that require less computational complexity. These edge points are essential for contour extraction and subpixel edge detection.
5. **Edge Orientation Enhancement:** A proven method for enhancing edge orientation, which involves applying a zero-phase lowpass filter to the sampled angles along the object’s contour. This is accomplished using the Kroon 5 × 5 kernel. This filter smooths the angle values on the edge without distorting the appropriate positional angles.
6. **Interest Points Selection:** Choose points where the measurement will be performed. This is typically based on the image’s attributes such as the edge angles, point locations, corners, features, etc.
7. **Subpixel Edge Detection:** Improve the location of points of interest in an image, predicated on a blurred edge model, which can be regressed using the Levenberg-Marquardt method. This process involves applying the presented sigmoidal model T(x), along with its corresponding partial derivatives, to arithmetically estimate the Jacobian. Additionally, location correction is performed by projecting the border to the normal line based on the enhanced orientations.
8. **Inverse projection:** The optimized edge points of interest, refined by subpixel edge detection, are projected from the image into the real world. This reverse projection employs both intrinsic and extrinsic calibrations that were previously estimated. Therefore, the quality of this projection relies on the accuracy of the estimated parameters. The part’s height and the calibration pattern’s thickness can be measured using a caliper, which usually presents no challenges.
9. **Neighborhood Fitting:** Implement curve fitting on neighboring edge points in real-world coordinates, in accordance with the expected shape of the analyzed section. The most frequently used shapes for curve fitting include lines, parabolas, and circles. Once the curve is fitted, it can be directly used to make the final measurements. Optionally, the edge points can be further refined by determining their closest location on the fitted curve, which can enhance the accuracy of the measurements.
10. **Measurement:** Compute the desired characteristics, e.g., radius and distance.

## 3. Results

### 3.1. Traceability Test

**Figure 8:**
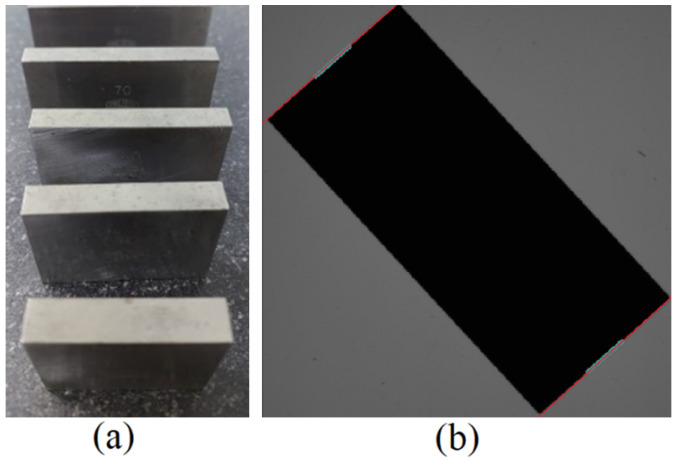
Gauge blocks used for the traceability test. (**a**) Actual image of the object. (**b**) Image used by the equipment, highlighting the sampled points and the regressed line.

Traceability validation was undertaken by measuring the gauge blocks under controlled conditions with traceable values, therefore assessing the accuracy of the systems, as shown in Figure 8. These blocks are grade A (+0.15 μm to −0.05 μm), with tolerances that are more stringent than those needed for the prototype.

Although the measurement faces of the gauge block are highly accurate, the same cannot be said for the other faces. The uncertainties observed on these faces did not exert a significant influence on the measurements. The dimensions of these faces were acquired using a calibrated caliper, and were used as height references in the measurement program.

Before conducting the test, the intrinsic parameters were ascertained by meticulously evaluating the calibration data to identify the most suitable set of parameters for accurately representing the checkerboard calibration patterns across different positions within the prototype’s measuring area. Similarly, the system’s extrinsic parameters were calibrated by positioning the checkerboard calibration pattern in the measuring area and adjusting for thickness. These calibrations were performed once before the experiments, therefore facilitating the measurement of all gauge blocks.

Measurement was conducted between the central points on the edges corresponding to the gauge block’s measuring faces. The location of the measurement points is enhanced using the proposed subpixel edge-detection technique. In this process, the line formed between the central point’s neighbors is exploited to optimize the results, as shown in Figure 8b.

Given the susceptibility of the captured images to noise, an examination was conducted to assess the impact of random noise on the final measurement results. This involved performing a sequence of measurements on a block in a static position, and evaluating the variation via standard deviation. After stacking the images, we observed a decrease in the variation of the results. However, no further significant reduction in variance was observed when the number of stacked images surpassed nine. The system’s variation, with stacking at the identified limit, is detailed in Table 1.

In the experiment, we sampled the dimensions between the measuring faces of blocks ranging from 40 to 80 mm. Each object was measured in ten different poses, and for each set, the means and standard deviations were calculated.

To test the method’s consistency with varying heights, two additional test sequences were conducted, by placing glass slides of known thickness between the calibrated reference planes and the parts. To adjust for the change in depth, the slide thickness was added to the Δz of the inverse projection part. In the second sequence, a glass slide of thickness 2.9 ± 0.03 mm was added, and in the third sequence, an additional glass slide of 2.92 ± 0.03 mm was added. The results are given in Table 2.

These findings reveal that the measurement system demonstrated a minimum accuracy of ±0.008 mm and repeatability (3σ) of ±0.019 mm when measuring gauge blocks in varying poses and heights. An examination of the test sequences showed a tendency towards increased measurements; however, this difference is negligible when contrasted to the result if the lengths were uncompensated for by the thickness of the added plates. This decrease in measurement accuracy can be attributed to the amplified uncertainty in depth caused by the added plates.

The monocular measurement system of the gauge block was run on a computer equipped with an i5-8300H processor and 16 GB of RAM, yielding an average processing time of 43.08 ms.

### 3.2. Comparative Test

**Figure 9:**
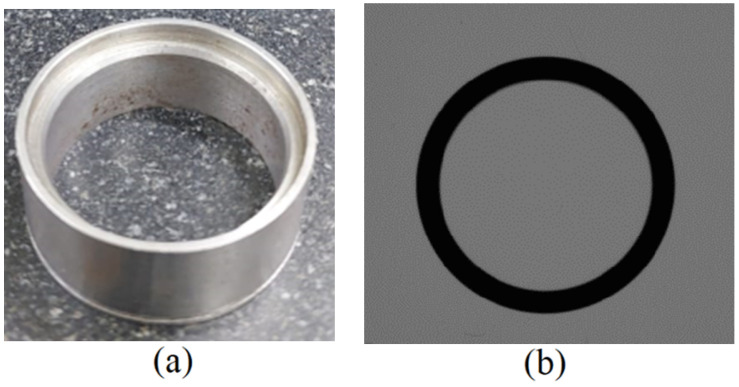
Sample used for comparison. (**a**) real image of the object (**b**) image captured by the prototype.

The prototype was juxtaposed with the Coordinate Measurement Machine (CMM), specifically the Mitutoyo B251 model, to measure the external profile of a specimen, as shown in Figure 9. This calibrated machine served as a reference, with a maximum anticipated error of 5+L/100 micrometers, where L signifies the distance traversed in millimeters.

60 points were sampled from the external contour of the part using the CMM. A nonlinear regression of these points was performed circularly to calculate the distance of each point from the center, therefore compensating for the probe size. This experiment was replicated three times, and the maximum, minimum, and average radii were ascertained for each iteration.

With the prototype, 1000 edge pixels were extracted from the image of the specimen’s outer profile. The positions of these edges were optimized using the subpixel technique, underpinned by the angle filter. To ensure consistency, the refined points were transposed into real-world coordinates via an inverse projection at 14.41 mm of measured height, applying the same calibration parameters and conditions as those of the gauge block. The refined edge points, in real-world coordinates, underwent additional processing with local circle fitting to improve determining their location. Ultimately, we performed a global regression of the circle samples, and the distance of each refined edge point from the center of the fitted circle was computed, akin to the CMM experiment. The findings are presented in Table 3.

Comparing the measurements obtained via both apparatuses shows that the proposed method showed similar circularity and slightly smaller dimensions than those of the reference equipment. A significant contributing factor to this difference is the presence of chamfers, which induce measurement deviations. This bias can be discerned in the measurements of the prototype in different poses, and can be compensated for since it remained constant across multiple measurements. Nonetheless, it is crucial to note that accuracy on the order of a thousandth of a millimeter is not expected from this device, given that calibration is performed using a checkerboard calibration pattern that carries a higher degree of uncertainty with it.

One comparative advantage of the prototype was its measuring speed. Despite manually capturing 60 precise measurements on the CMM taking several minutes, the prototype could measure 1000 points within an average processing time of 145.70 ms. Another advantage is its ability to execute measurements without contact nor requiring that components be secured, therefore minimizing measurement interferences. As a drawback, image-based measurements lack flexibility om measuring varying depths and in occluded regions, and regular lenses might introduce bias into the measurements, brought on by the profile of the outer side.

## 4. Discussion

In this paper, a system was calibrated using a planar checkerboard pattern and analyzed using two types of tests. The results show that the prototype maintains traceability across different gauge block sizes and positions. Additionally, when glass plates were added and the height varied, the system showed consistent properties at different heights, demonstrating its effectiveness in compensating for object height and tilt. In the comparative tests, the system successfully matched the measurements of a circular sample within the expected tolerances levels, when compared to a calibrated alternative machine. These tests validate the suitability of the current photogrammetry and computer vision methods, along with the setup for the evaluated samples. Furthermore, the algorithm demonstrated satisfactory processing time, making efficient use of computer resources through direct implementation in C++.

When comparing this study with related works, our proposed method, which applies monocular vision to determine the dimensions of mechanical parts, shows promising results. In this study, the proposed method achieved a maximum mean error of 0.008 mm and a standard deviation of 0.0063 mm while measuring straight sections on gauge blocks. These reported results are presented in Table 4.

By comparison, Guo and Fu [1], used a simple geometric approach for planar measurements, and tested a 25 mm coin and 35 mm and 85 mm block, which resulted in an accuracy of 0.028 mm, 0.075 mm, and 0.196 mm, and standard deviations in sequential readings at 0.0011 mm, 0.0043 mm, and 0.0083 mm, respectively. Moreover, Li [16] employed a monocular vision system to measure the height and radius of the cylindrical parts of shafts. Although the author did not determine the average deviation, the standard deviation was 0.01 mm for the radius and 0.009 mm for height. Furthermore, TAN et al. [17] employed a combination of monocular vision and a laser line projected in a structured light scheme to measure the radius of shaft portions on three axes. Their study resulted in accuracies at 0.013 mm, 0.011 mm, and 0.015 mm, and mean standard deviations at 0.015 mm, 0.006 mm, and 0.005 mm. Finally, Sun et al. [10] developed a structured light system using three lasers to analyze objects on a rotating plate for three-dimensional reconstruction. They evaluated their design with 40, 50, 60, and 70 mm gauge blocks and obtained average errors at 0.025 mm, 0.006 mm, 0.006 mm, and 0.03 mm, and standard deviations at 0.1099 mm, 0.1056 mm, 0.1007 mm, and 0.0856 mm, respectively. Although the last approach could reproduce more complex parts with less ground truth, it showed the relevance of simpler schemes for measuring parts with only plane dimensions.

Based on the results of this study, as well as the results of other relevant studies, the proposed prototype demonstrated satisfactory performance. Similar to other approaches, the proposed method employs subpixel edge-detection techniques and accounts for lens distortions and plane misalignment. However, unlike other applications that used different edge models and conventional filters or neighborhood fitting to estimate the normal direction of an edge pixel, our study employed the edge model and the Kroon filter in combination with a zero-phase filter to improve angle accuracy. The neighborhood fitting was only applied after the lens distortion was corrected and mapped to the 3D world coordinates. Furthermore, the prototype adopted a formulation to compensate for the measuring height.

The limitations associated with the prototype assume that planar measurements are parallel to the calibrated plane since depth cannot be determined using a single camera. Second, the regular lens introduced limitations in terms of perspective and the edge depth profile. Lastly, the problem as it was framed confined the objective to measuring dimensions between elements on the outer contour of the parts.

Given the current limitations, the system could be further enhanced. One valuable enhancement would involve incorporating an automated method for estimating the object height, reducing reliance on ground truth measurements. Regarding applicability, the prototype machine can be upgraded with feature additions and adjustments to measure a wider range of mechanical parts, expanding versatility beyond these initially tested applications. It could effectively assess various planar mechanical parts, including but not limited to rings, bearings, connections, shafts, plates, gears, cams, linkages, and brackets.

To effectively implement the prototype in industry, one must consider optimizing the design to enhance the device’s robustness, allowing it to withstand industrial environments and meet industry standards for certifications and compliance. This requires testing, validations, and design documentation, as well as establishing maintenance procedures to ensure the proper upkeep and longevity of the prototype. Furthermore, one must establish a supply chain to ensure cost-effective inventory management. Along these lines, collaborating with industry partners could be important to gather information on use cases and applications. Additionally, rigorous quality control measures and effective marketing strategies are vital for establishing a strong customer base. It is important to note that despite these additional investments, the evidence suggests that the system can consistently maintain its cost-effectiveness advantage over other measurement methods currently used by industries. This is usually due to the costs associated with these methods, which may include labor costs and waste resulting from human error. By implementing the measurement method proposed in this article, these avoidable costs could be significantly reduced.

Despite the aforementioned initial investments, the cost-effectiveness of the prototype was achieved via a strategic combination of factors, first, by designing solutions without moving parts, eliminating expenses associated with intricate mechanical components, thus leading to reduced manufacturing and maintenance costs. Second, we used widely available entry-level industrial vision equipment also allowing cost savings by leveraging affordable off-the-shelf hardware, thus making the prototype more accessible and scalable. Additionally, open-source libraries further enhanced the cost-effectiveness since this eliminated the need for expensive proprietary software licenses, meaning that commercial applications can be undertaken without any additional charges. Collectively, these measures ensured a financially viable solution.

Compared to the conventional manual measuring method using calipers, this prototype offers similar precision, while also providing advantages such as offering no-contact, faster, and less human-error-prone measurements. Additionally, integrated machine vision systems overcome difficulties faced by manual methods in determining certain characteristics e.g., the largest diameter, the smallest diameter, or the roundness of a corner. These machine vision systems can analyze multiple characteristics from a single image. Although the prototype may require a higher initial cost, it becomes cost-effective when considering the meticulousness and time required for manual measurements.

This system offers several advantages over existing computer vision systems designed for the same purpose. First, it incorporates straightforward industrial components, while guaranteeing dependability, compatibility, and cost-efficiency. Furthermore, the system is transparent and flexible, since details on the methods and algorithms used were provided. It also enhances accessibility and affordability by incorporating regular lenses, therefore enabling easy deployment across various environments. Compared to machine vision systems with longer focal length lenses, a fixed-focus lens with shorter focal lengths offers a broader field of view and greater depth of field. As this prototype shows, this system can accurately measure parts within a 100 × 100 mm area via a single image. By eliminating intricate motion mechanisms, the system is simpler, more efficient, and more robust.

In terms of practical applications, this system has proven to be effective in maintaining traceability across diverse poses and heights, ensuring consistent and reliable results across a variety of scenarios. These attributes make it well-suited for evaluating comparable parts or fine-tuning manufacturing processes, e.g., conformity verification. In summary, this system broadens the potential uses of computer vision systems, particularly in situations where the cost or agility of existing systems are restrictive.

## 5. Conclusions

This paper proposes a method for automatically measuring specifications on the external contours of mechanical parts using image processing. This setup uses low-cost equipment e.g., an entry-level industrial camera, ordinary lenses, and backlight. An analysis of the topic is provided here, with a functional prototype containing procedures and adjustments being derived from literature. This prototype has been validated via both traceability and comparative tests, demonstrating its ability to perform measurements quickly and accurately in either straight or circular sections. When making planar measurements of mechanical parts, the perceived standard deviation was 0.0063 mm, and accuracy was 0.008 mm. The applications of the method developed here are promising with respect to solving widely employed industry problems inherent to making manual measurements. Relative to manual measurements, this prototype offers some improvements, including removing human bias from caliper measurements, preventing errors caused by distortions generated by contacting the parts, and increasing measurement speeds, meaning that many manufactured components can be measured quickly. Additionally, the system can be further improved using higher-grade instruments and expanding the measuring model to represent known elements, e.g., chamfers.

## Figures and Tables

**Table 1:** Observed deviation resulting from random image noise in repetitive measurements.

Gauge Block	3σ (mm)
70 mm	0.004

**Table 2:** Measuring the Gauge Block in arbitrary poses, within the constraints, using the proposed prototype.

Gauge Block		Height (mm)	Sequence 1 (mm)		Sequence 2 (mm)		Sequence 3 (mm)	
size	uncertainty	Δz	μ	3σ	μ	3σ	μ	3σ
40 mm	<0.15 µm	8.98 ± 0.02	39.998	0.01	39.995	0.005	40.005	0.008
50 mm	<0.15 µm	8.96 ± 0.02	49.997	0.009	49.995	0.009	50.004	0.009
60 mm	<0.15 µm	8.88 ± 0.02	59.992	0.006	59.997	0.006	60.000	0.008
70 mm	<0.15 µm	8.98 ± 0.02	69.998	0.019	70.000	0.006	70.002	0.009
80 mm	<0.15 µm	8.92 ± 0.02	80.000	0.012	80.006	0.008	80.007	0.009

**Table 3:** Measuring the radius profile of a cylindrical sample in mm to validate the prototype.

Sample Set	Mitutoyo B251			Proposed Method		
	Mean	Min.	Max.	Mean	Min.	Max.
1	18.025	18.021	18.028	18.015	18.011	18.019
2	18.025	18.020	18.029	18.017	18.012	18.021
3	18.025	18.022	18.029	18.017	18.012	18.022
4	-	-	-	18.017	18.012	18.024
5	-	-	-	18.015	18.011	18.020
$\bar{X}$	18.025	18.021	18.029	18.016	18.012	18.021

**Table 4:** Mean error and standard deviation, in mm, comparison between method of planar dimension measurement using monocular vision.

	Proposed	Li [16]		TAN et al. [17]			Sun et al. [10]			
	Max. Deviation	Radius	Height	Shaft1	Shaft2	Shaft3	Gauge 40 mm	Gauge 50 mm	Gauge 60 mm	Gauge 70 mm
Mean Dev.	0.008	-	-	0.013	0.011	0.015	0.025	0.006	0.006	0.03
STD	0.0063	0.01	0.009	0.015	0.006	0.005	0.085	0.1007	0.1056	0.1099

## Data Availability

The data used to support the findings of this study are available from the corresponding author upon request.

## References

[1] Guo Q., Fu W. (2020). An improved measurement method of size of mechanical parts based on monocular vision. J. Phys. Conf. Ser..

[2] Cox D.R. (2009). Randomization in the design of experiments. Int. Stat. Rev..

[3] Dang A.-T., Hsu Q.-C., Truong T.-T. (2021). A simple method for dimensional measurement of ring-shaped objects using image processing technique. Int. J. Adv. Manuf. Technol..

[4] Ren Z., Fang F., Yan N., Wu Y. (2022). State of the art in defect detection based on machine vision. Int. J. Precis. Eng. Manuf.-Green Technol..

[5] Hornberg A. (2006). Handbook of Machine Vision.

[6] Guerra M.G., Galantucci L.M., Lavecchia F., De Chiffre L. (2021). Reconstruction of small components using photogrammetry: A quantitative analysis of the depth of field influence using a miniature step gauge. Metrol. Meas. Syst. Polska Akademia Nauk.

[7] Jiang R., Jáuregui D.V., White K.R. (2008). Close-Range Photogrammetry Applications in Bridge Measurement: Literature Review. Measurement.

[8] Percoco G., Lavecchia F., Salmerón A.J.S. (2015). Preliminary Study on the 3d Digitization of Millimeter Scale Products by Means of Photogrammetry. Proceded CIRP.

[9] Elkhrachy I. (2021). Accuracy assessment of low-cost unmanned aerial vehicle (uav) photogrammetry. Alex. Eng. J..

[10] Sun Q., Ren Z., Zhu J., Dai W., Wang M., Sun M. (2022). A Three-Dimensional Structured Light Vision System by Using a Combination of Single-Line and Three-Line Lasers. Sensors.

[11] Karsznia K., Osada E. (2022). Photogrammetric precise surveying based on the adjusted 3d linear control network deployed on a measured object. Appl. Sci..

[12] Sun Q., Hou Y., Tan Q., Li G. (2013). A planar-dimensions machine vision measurement method based on lens distortion correction. Sci. World J..

[13] Duan Z., Wang N., Fu J., Zhao W., Duan B., Zhao J. (2018). High precision edge detection algorithm for mechanical parts. Meas. Sci. Rev..

[14] Lee S.W., Lee S.Y., Pahk H.J. (2018). Precise edge detection method using sigmoid function in blurry and noisy image for tft-lcd 2d critical dimension measurement. Curr. Opt. Photonics.

[15] Fabijańska A. (2012). A survey of subpixel edge detection methods for images of heat emitting metal specimens. Int. J. Appl. Math. Comput. Sci..

[16] Li B. (2018). Research on geometric dimension measurement system of shaft parts based on machine vision. EURASIP J. Image Video Process..

[17] Tan Q., Kou Y., Miao J., Liu S., Chai B. (2021). A model of diameter measurement based on the machine vision. Symmetry.

[18] Aleixo F., O’Callaghan S.A., Ducla Soares L., Nunes P., Prieto R. (2020). Aragoj: A free, open-source software to aid single camera photogrammetry studies. Methods Ecol. Evol..

[19] Li X., Liu W., Pan Y., Ma J., Wang F. (2019). A knowledge-driven approach for 3d high temporal-spatial measurement of an arbitrary contouring error of cnc machine tools using monocular vision. Sensors.

[20] Xu S., Liu M. Feature selection and pose estimation from known planar objects using monocular vision. Proceedings of the 2013 IEEE International Conference on Robotics and Biomimetics (ROBIO).

[21] Noonan J., Rotstein H., Geva A., Rivlin E. (2019). Global monocular indoor positioning of a robotic vehicle with a floorplan. Sensors.

[22] Li Y., Liu X.L., Xu D., Zhang D.P. (2020). Orientation measurement for objects with planar surface based on monocular microscopic vision. Int. J. Autom. Comput..

[23] Kroon D. (2009). Numerical Optimization of Kernel Based Image Derivatives.

[24] An G.H., Lee S., Seo M.W., Yun K., Cheong W.S., Kang S.J. (2018). Charuco board-based omnidirectional camera calibration method. Electronics.

[25] Hartley R., Zisserman A. (2003). Multiple View Geometry in Computer Vision.

[26] Szeliski R. (2011). Computer Vision: Algorithms and Applications.

[27] Zhang Z. Flexible camera calibration by viewing a plane from unknown orientations. Proceedings of the Seventh IEEE International Conference on Computer Vision.

[28] Ye J., Fu G., Poudel U.P. (2005). High-accuracy edge detection with blurred edge model. Image Vis. Comput..

[29] Nalwa V.S., Binford T.O. (1986). On detecting edges. IEEE Trans. Pattern Anal. Mach. Intell..

[30] von Gioi R.G., Randall G. (2017). A sub-pixel edge detector: An implementation of the canny/devernay algorithm. Image Process. Online.

[31] Farrell J., Xiao F., Kavusi S. (2006). Resolution and light sensitivity tradeoff with pixel size. Digital Photography II.

[32] Losson O., Macaire L., Yang Y. (2010). Comparison of color demosaicing methods. Adv. Imaging Electron Phys..

